# The Effect of Pulmonary Hypertension on Renal Function Dynamics in Left-Heart Failure Patients

**DOI:** 10.3390/biomedicines13030684

**Published:** 2025-03-10

**Authors:** Robert Dragu, Adrian Abramovici, Kasem Abu Zeid

**Affiliations:** 1 Internal Medicine C Department, Galilee Medical Center, Nahariya 22100, Israel; 2 Pulmonary Vascular Diseases Service, Galilee Medical Center, Nahariya 22100, Israel

**Keywords:** pulmonary hypertension, heart failure, renal function, cardiorenal syndrome

## Abstract

**Objectives:** Cardiorenal syndrome (CRS) is a complex disorder characterized by concurrent dysfunction of the heart and kidneys, with their detrimental effects perpetuating a bidirectional cycle. This study aimed to examine the clinical and hemodynamic factors associated with changes in renal function in patients with pulmonary hypertension (PH) secondary to chronic heart failure (HF). **Methods:** A total of 108 patients with HF were evaluated using right-heart catheterization. **Results:** 75 patients (69.4%) were diagnosed with PH. The mean baseline estimated GFR (beGFR) was similar in noPH (64 ± 21 mL/min/1.73 m^2^) and PH group (63 ± 23 mL/min/1.73 m^2^) (*p* = 0.71). After a median follow-up of 7 months, the last eGFR (leGFR) in the noPH and PH groups was comparable (49 ± 24 vs. 52 ± 25 mL/min/1.73 m^2^ respectively; *p* = 0.62). However, in the PH group, for patients with baseline Cr (bCr) < 1.5 mg/dL, the reduction in eGFR showed a graded inverse relationship to serum creatinine, as compared with bCr ≥ 1.5 mg/dL, for whom beGFR and leGFR demonstrated large overlap. In a multivariable regression analysis, the primary independent predictors of leGFR were baseline creatinine, age, diabetes mellitus, left ventricular ejection fraction below 45%, and use of mineralocorticoids antagonists. The model explained 66% of the variance in leGFR. **Conclusions:** In a cohort of left HF and PH, an inverse non-linear and graded association between the baseline serum creatinine levels and the variation in estimated GFR was demonstrated, contrary to those without PH, for whom this relationship was linear and constant. The distinct patterns of GFR decline influenced by age, low ejection fraction, diabetes, and mineralocorticoid underscore the need for individualized treatment strategies.

## 1. Introduction

Chronic left-heart failure (HF) is a major contributor to pulmonary hypertension (PH), representing one of its most common forms [1]. The presence of PH in this context is associated with an unfavorable prognosis [2,3,4,5,6]. In HF, PH arises primarily from increased left ventricular (LV) filling pressures, which subsequently elevate pulmonary capillary wedge pressure (PCWP). Cardiorenal syndrome (CRS) is a complex disorder involving simultaneous dysfunction of the heart and kidneys, perpetuating a bidirectional cycle of adverse effects [7].

The consensus conference of the Acute Dialysis Quality Initiative identified and classified cardiorenal syndrome into five distinct types [8]. Per this classification, the decline in renal function observed in patients with heart failure may be categorized as cardiorenal syndrome (CRS) type 2 and/or type 4.

A number of mechanisms have been proposed as involved in CRS type 2, including central venous pressure elevation causing reduced arteriovenous pressure gradient across the kidney, renin–angiotensin–aldosterone system activation, sympathetic over-activity, and oxidative injury on endothelial function [9].

The connection between CRS and PH is reinforced by evidence indicating that right atrial pressure correlates more strongly with kidney dysfunction than with cardiac index in PAH patients [10]. Similar findings in group 2 PH patients further underscore the critical role of venous congestion in impairing renal function [11,12]. Findings from patients with various types of heart failure support this observation, identifying elevated right-sided filling pressures as the leading determinant of kidney function decline [12].

The aim of the present study is to explore renal function dynamics in patients with HF and PH, as compared to those without PH, and to identify clinical, echocardiographic and/or hemodynamic predictors of these interactions. This study also investigated how baseline serum creatinine levels influenced variations in glomerular filtration rate during extended follow-up periods.

## 2. Materials and Methods

### 2.1. Patients

The study involved patients with heart failure (HF) and NYHA functional capacity of 2 or higher, who had right heart catheterization (RHC) between 1 May 2016, and 31 December 2022. The exclusion criteria encompassed individuals below the age of 18, those experiencing acute decompensated heart failure (HF), alongside any identified or potential alternative causes of pulmonary hypertension (PH) aside from HF. These causes included congenital heart disease, valvular diseases, lung diseases, connective tissue disorders, and hematological conditions.

This study followed the Declaration of Helsinki guidelines and was approved by Galilee Medical Center’s institutional review board.

### 2.2. Hemodynamic Assessment

Pressure measurements during catheterization were performed with patients in a resting supine position, following standard guidelines and using fluoroscopic assistance. Pressures were measured with a 7F Swan-Ganz catheter, cardiac output (CO) was determined through the Fick method, and recordings were made at end expiration. Pulmonary vascular resistance (PVR) was calculated using established equations. Based on hemodynamic assessments, patients were allotted into two groups: (i) no PH, defined as mPAP ≤ 20 mmHg, and (ii) PH, characterized by wedge pressure above 15 mmHg and mPAP > 20 mmHg.

### 2.3. Echocardiography

The systolic function of the right ventricle (RV) was evaluated quantitatively using tricuspid annulus plane systolic excursion (TAPSE) and qualitatively through visual assessment. Moderate or greater impairment was classified as right ventricular systolic dysfunction. Echocardiographic and hemodynamic assessments were conducted during the same hospital stay.

### 2.4. Assessment of Renal Function

Baseline serum creatinine (bCr) levels were measured from venous blood samples collected upon admission during the index hospitalization for RHC. The calculation of baseline glomerular filtration rate (beGFR) utilized the abbreviated methodology described in the Modification of Diet in Renal Disease study [13]. Last estimated GFR (leGFR) and supplementary laboratory data were obtained from medical records by chart review. The difference (deGFR) between baseline and last estimated GFR was calculated.

### 2.5. Statistics

Continuous data are reported as mean ± standard deviation (SD) or medians with interquartile ranges (IQRs), whereas categorical data are summarized as counts and percentages. Baseline group characteristics were analyzed using an analysis of variance (ANOVA) for continuous parametric variables. Non-parametric variables were analyzed using a Kruskal–Wallis test, while a chi-square (χ^2^) analysis was used for categorical variables.

It has been demonstrated that kidney function declines non-linearly [14], rendering traditional linear models insufficient for capturing its complexity.

Restricted cubic spline transformations [15] were applied to the continuous independent variable to identify potential non-linear relationships between bCr and the predicted probability of deGFR magnitude. Marginal effects were employed to calculate the variation in deGFR corresponding to unit changes in bCr, with the aim of identifying significant alterations in the slope of the spline [16].

To minimize confounding, we employed propensity score matching (PSM). Propensity scores were calculated using logistic regression, incorporating demographic, clinical, and hemodynamic variables. PH patients were matched to noPH patients using a nearest-neighbor algorithm with a caliper of 0.2, resulting in balanced groups for comparison. Post-matching balance was assessed using standardized mean differences, ensuring all covariates had differences below 0.1, indicating adequate balance.

The association between potential predictors and leGFR was determined by fitting univariate linear regression models. Variables deemed clinically significant or with *p* < 0.1 in univariable analysis were entered into a backward-selected regression model. Variables considered for inclusion in the models were as follows: age, LV systolic function, PH, RV systolic function, aortic mean pressure, right atrial pressure, baseline serum creatinine, gender, history of diabetes mellitus and/or hypertension, use of loop diuretics and/or use of mineralocorticoids antagonist, administration of angiotensin-converting enzyme inhibitors or angiotensin receptor blockers. Statistical significance was defined as a two-sided *p*-value of <0.05. Analyses were carried out using SPSS 15.0 (Chicago, IL, USA) and STATA 12.0 (STATA Corp., College Station, TX, USA).

## 3. Results

Out of 127 patients with chronic left-sided HF who underwent RHC during the study period, 19 were excluded due to missing baseline creatinine data. The final study population comprised the remaining 108 subjects, with 75 (69.4%) of them having PH. The mean beGFR was similar in the no-PH group (64 ± 21 mL/min/1.73 m^2^) and the PH group (63 ± 23 mL/min/1.73 m^2^) (*p* = 0.71).

Table 1 outlines the clinical and hemodynamic profiles of the study population, categorized by the occurrence of PH. Individuals in the PH group exhibited higher levels of PCWP, RAP, and PVR. Most patients in both cohorts exhibited preserved left ventricular systolic function, whereas right ventricular dysfunction was more frequently observed among those diagnosed with pulmonary hypertension. No differences were noted between the groups as concerns the number of patients treated by loop diuretics.

The median follow-up duration was 7 months (interquartile range: 5–9 months). At the end of this period, a reduction in GFR was observed, with leGFR in the noPH and PH groups being similar (49 ± 24 vs. 52 ± 25 mL/min/1.73 m^2^, respectively; *p* = 0.62). The BUN to serum creatinine ratio assessed diuretic treatment intensity. There were no significant differences observed between the noPH and PH groups at baseline (20.0 ± 6.9 vs. 21.9 ± 8.0; *p* = 0.23) or at follow-up (21.0 ± 6.2 vs. 24.2 ± 9.0; *p* = 0.10).

Table 2 presents the outcomes of a multivariable regression analysis, examining predictors of leGFR as a continuous parameter, within both noPH and PH groups. Key independent predictors included baseline creatinine, age, diabetes mellitus, left ventricular ejection fraction (LVEF) below 45%, and use of mineralocorticoids antagonists. This model explained 66% of the variance in leGFR.

### 3.1. Predictive Model for Renal Function Deterioration

**Figure 1:**
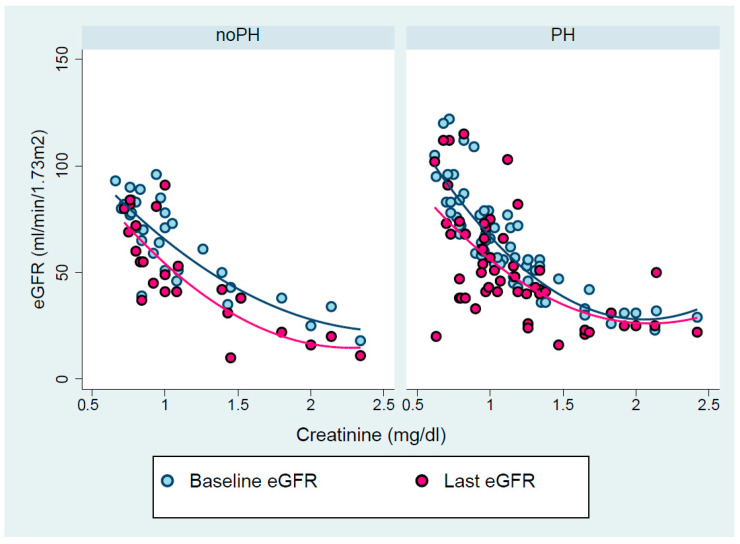
Scatter plot and fit lines of baseline creatinine vs. baseline and last eGFR in patients with left HF without and with PH.

The hyperbolic relationships between bCr and both beGFR and leGFR, according to PH presence, are depicted in Figure 1. In the no-PH group, the dispersion pattern of deGFR is homogenous over the entire creatinine spectrum. However, in the PH group, for bCr < 1.5 mg/dL, deGFR showed a graded inverse relationship to serum creatinine, as compared with bCr ≥ 1.5 mg/dL, for whom beGFR and leGFR demonstrated a large overlap.

**Figure 2:**
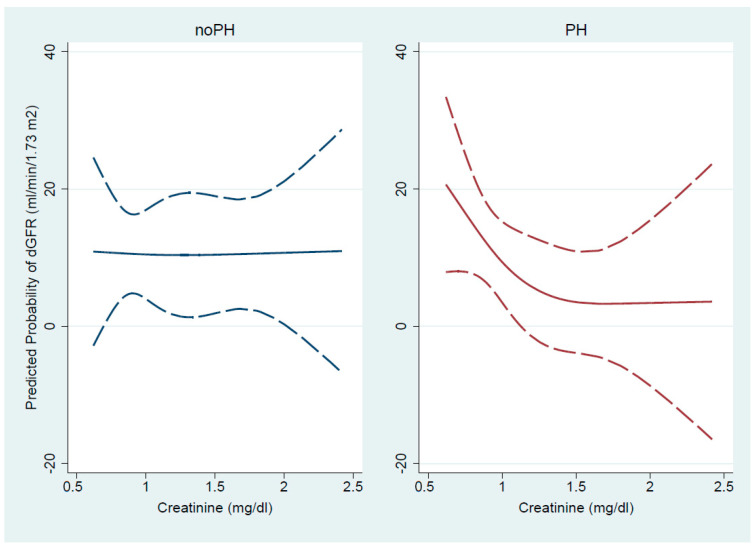
Cubic spline analysis of the probability of variation in estimated GFR in left HF according to presence of PH. The dash dot lines represent the 95% confidence interval.

Cubic spline transformation was applied to investigate the possible non-linear relationship between bCr and deGFR. In the no-PH group, it was observed that the predicted probability of change in GFR magnitude was linear and constant over the entire bCr range, whereas in the PH group, deGFR decreased progressively with creatinine increment, in a J-shaped curve. The predicted mean of deGFR probability enabled the detection of a change in the spline’s slope, which became steeper for patients with bCr levels below 1.5 mg/dL (Figure 2).

### 3.2. Propensity Score Analysis

After PSM, each group consisted of 33 patients, with baseline characteristics being well balanced. Post-matching, leGFR in the PH group was similar to that in no-PH group (52.43 ± 16.47 vs. 56.96 ± 16.47 mL/min/1.73 m^2^, respectively; *p* = 0.50).

In the reanalyzed multivariable regression model for the matched cohort, baseline creatinine, age, LVEF < 45%, and use of mineralocorticoids antagonists were identified again as significant predictors of renal function decline, while diabetes mellitus was not statistically significant, potentially due to reduced sample size post-matching (Table 3).

### 3.3. Subgroup Analysis

A subgroup analysis model was developed that included previously identified independent predictors, such as baseline creatinine, age, LVEF < 45%, and MRA use. Additionally, RV dysfunction was incorporated into the regression models to examine its potential role in predicting renal function decline. This analysis was performed separately for patients with and without diabetes mellitus.

**Figure 3:**
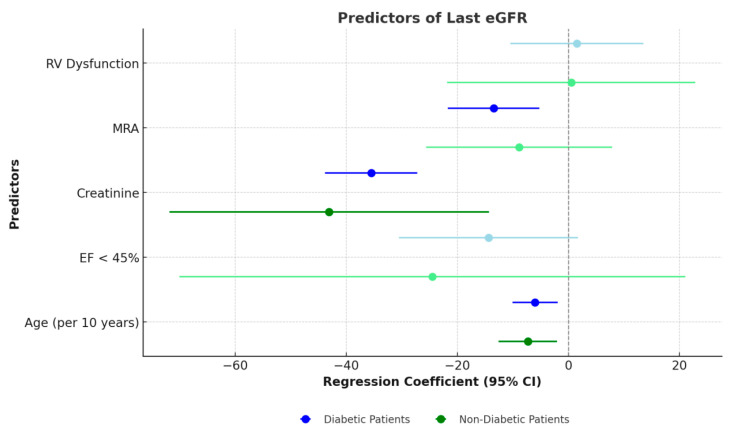
Forest plot showing regression coefficients and 95% confidence intervals for predictors of last eGFR in diabetic and non-diabetic subgroups.

The results, as illustrated in Figure 3, identify significant predictors and highlight differences between diabetic and non-diabetic subgroups. For patients with diabetes, age, baseline creatinine levels, and the use of MRA were found to be significant predictors of renal function. However, LVEF < 45% and RV dysfunction did not achieve statistical significance. In contrast, for non-diabetic patients, the significant predictors were age and baseline creatinine, whereas MRA, EF < 45%, and RV dysfunction did not demonstrate statistical significance. The models explained 71% of the variance in renal function outcomes for diabetic patients, compared to 52% for non-diabetic patients.

## 4. Discussion

Our findings indicate a similar decline in renal function among patients with left-sided HF, regardless of the presence of pulmonary hypertension.

Baseline serum creatinine, age, diabetes mellitus, left ventricular ejection fraction below 45%, and use of mineralocorticoid antagonist were independent predictors of renal function at the end of the follow-up period and explained two-thirds of the variability of leGFR.

An inverse non-linear and graded association between the baseline serum creatinine levels and the probability of change in estimated GFR was demonstrated in PH patients, contrary to those without PH, for whom this relationship was linear and constant.

In the normal aging population, a reduction in the estimated GFR of 0.5 to 1.0 mL/min/1.73 m^2^/year is to be expected, while CKD patients may present a more marked deterioration [17]. Studies on HF and CKD cohorts reported rapid estimated GFR decline, ranging between 10 and 20% at an up to 1-year follow-up [18,19].

Our finding corroborates previous studies which established that the HF population shows a high prevalence of chronic kidney dysfunction and a graded progression of renal function worsening [8,20,21,22]. The mechanisms through which HF contributes deleteriously to kidney function reduction include renal venous congestion, reduced renal perfusion, and activated hormonal and inflammatory pathways. The Cardiovascular Health Study demonstrated that elderly individuals in the highest NT pro-BNP quartile faced a greater risk of rapid GFR decline compared to those in the lowest quartile [23], suggesting that subclinical or biomarker-based diagnoses of HF are linked to progressive renal function deterioration.

The heart and kidneys interact bidirectionally, with declining GFR aggravating HF and vice versa. Older age and diabetes mellitus are risk factors for HF and chronic kidney disease [22], and not surprisingly, in the present study, they have been demonstrated as predictors for renal function deterioration. In symptomatic as well as asymptomatic systolic HF, renal dysfunction was found strongly associated with outcomes [20,24,25], supporting our finding that an LV ejection fraction under 45% represents a prognostic factor for progressive loss of kidney function.

Previous studies have shown that the use of mineralocorticoid antagonist, as part of the optimal medical therapy for HF, was correlated to renal function reduction [26]. This observation was similarly evident in the current study, where our findings demonstrated significant negative impact of mineralocorticoid antagonist on kidney function.

The results highlight notable differences between diabetic and non-diabetic patients in the predictors of renal function decline. Baseline creatinine and age consistently impacted eGFR across both groups, underscoring their universal relevance in cardiorenal syndromes. However, the significant negative association of MRA with eGFR in diabetic patients emphasizes the need for tailored therapeutic strategies in this subgroup, and it is in line with the study of Matsumoto [27].

Left-sided heart-failure-induced pulmonary hypertension reflects a progressed stage of the condition and is correlated with elevated mortality rates. Despite the multifaceted nature of its pathobiology, the prognosis largely relies on right ventricular function [28,29,30]. Under normal circumstances, the RV operates within a vascular system characterized by low resistance and high capacitance. However, its thin-walled structure makes it ill-equipped to manage elevated pulmonary arterial pressure, resulting from the backward transmission of increased pulmonary capillary wedge pressure. Prolonged exposure to a high afterload prompts the RV to adapt initially through hypertrophy, followed by ventricular dilation, akin to the remodeling seen in pulmonary arterial hypertension. The ultimate outcome of this remodeling process is systolic and/or diastolic dysfunction of RV, translated into elevated RA pressure.

Renal congestion is now acknowledged as a possible factor contributing to CRS type 2, and not only is its presence de rigueur in systemic congestion, but it portends prognostic information [12,31,32]. Starting with studies on animals [33] and followed by investigations on human acute decompensated and chronic heart failure, it has been shown that renal congestion is an independent factor linked to the deterioration of kidney function [11,12,34,35]. Despite the lack of homogeneity in population characteristics, the congruent factor found independently associated with renal dysfunction was increased central venous pressure. The backward transmission of this pressure to renal veins impose modifications in intra-renal hemodynamics, with consequences on both ultrafiltration pressure and hydrostatic interstitial pressure, with GFR being influenced by the balance of them [36]. An increase in renal vein pressure triggers an increase in the ultrafiltration pressure, which is normally corrected by intrinsic kidney autoregulation [36,37]. However, this rectification is lost in cases of chronic kidney disease [38], probably explaining our findings that the subjects suffering from PH due to left HF and a creatinine > 1.5 mg/dL demonstrated preserved GFR during the follow-up, in light of increased ultrafiltration pressure. In contrast, a serum creatinine below 1.5 mg/dL, entailing an adequate renal autoregulation, makes the elevated intra-renal interstitial pressure predominant over the balance and, through the compression of tubules, imposing a reduced GFR.

Despite the hypothesized role of the right ventricle in renal function dynamics, its dysfunction did not emerge as a significant predictor in the present analysis. This may be attributed to the multifactorial nature of renal impairment in left-heart failure. Factors such as systemic hemodynamics, neurohormonal activation, and intrinsic renal mechanisms likely overshadow the direct impact of RV dysfunction.

Other mechanisms such as tubule–glomerular feedback, colloid osmotic pressure, or neurohormonal systems may also be involved in the nested apparatuses that contribute to GFR reduction in subjects with HF. However, the failing kidney is characterized by beneficial adaptations to restore GFR, mechanisms which later became detrimental and induce declining renal function.

The decline in GFR unfolds in distinct patterns, influenced by factors such as age, baseline creatinine, low ejection fraction, diabetes, and mineralocorticoid use. These variations underscore how patients experience kidney function deterioration uniquely, each shaped by their individual risk profiles. The differential impact of MRA in diabetic patients raises concerns about their potential nephrotoxicity or heightened sensitivity in this population, calling for close monitoring of renal function in diabetic patients receiving MRA. Therefore, embracing personalized medicine is essential—tailoring treatment strategies to each patient’s unique combination of factors to effectively manage and slow the progression of renal impairment.

## 5. Study Limitations

This study has several limitations. First, its retrospective nature inherently limits the ability to establish causal relationships. While our findings provide meaningful insights into the interplay between PH, renal function, and hemodynamics in HF patients, prospective studies are needed to validate these associations and explore causal mechanisms further.

Second, hemodynamic data were collected only at baseline, and follow-up invasive assessments were not available. Routine clinical practice does not typically include serial right-heart catheterizations due to their invasive nature, associated risks, and ethical considerations. While this limits the ability to track hemodynamic changes over time, the hemodynamic parameters included in this study remain crucial for understanding baseline disease severity and its relationship with renal function.

Third, the sample size was reduced after propensity score matching. Although this matching process inherently leads to a smaller cohort, it strengthens internal validity by minimizing baseline differences between groups. Importantly, despite the reduced sample size, our results remained consistent with those from the full cohort, reinforcing the robustness of our findings.

Finally, our analysis was restricted to available clinical and hemodynamic parameters, meaning residual confounding from unmeasured variables cannot be entirely excluded. Future studies incorporating additional biomarkers and longitudinal hemodynamic assessments may further refine our understanding of these interactions.

## 6. Conclusions

In a cohort of left HF and PH, an inverse non-linear and graded association between the baseline serum creatinine levels and the variation in estimated GFR was demonstrated, contrary to those without PH, for whom this relationship was linear and constant. Baseline serum creatinine, age, diabetes mellitus, left ventricular ejection fraction below 45%, and use of mineralocorticoid antagonist were independent predictors of renal function at the end of the follow-up period. Future research is necessary to determine the impact of these findings on therapeutic approach in this population.

## Figures and Tables

**Table 1:** Comparison of clinical and hemodynamic characteristics in chronic left-heart failure patients grouped by pulmonary hypertension status.

Characteristics	No PH	PH	*p*-Value
	n (33)	n (75)	
Age (years)	72.0 ± 9.1	68.7 ± 13.2	0.19
Female (%)	18 (54.5)	56 (74.6)	0.88
Diabetes Mellitus (%)	19 (57.5)	37 (49.3)	0.92
Hypertension (%)	25 (75.7)	51 (68.0)	0.65
eGFR (mL/min/1.73 m^2^)	64 ± 21	63 ± 23	0.11
Hb (g/dL)	11.8 ± 1.9	11.6 ± 2.4	0.76
LVEF < 45%	2 (6.7)	8 (10.6)	0.38
RV dysfunction	2 (6.7)	22 (29.3)	0.005
Hemodynamic variables			
PCWP (mmHg)	11.0 ± 4.3	18.9 ± 6.9	<0.0001
mPAP (mmHg)	16.4 ± 3.3	37.8 ± 10.9	<0.0001
SV (mL)	56.7 ± 13.7	59.6 ± 22.1	0.51
CO (L/min)	4.2 ± 1.0	4.5 ± 1.5	0.23
Aortic mean pressure (mmHg)	95.9 ± 14.2	97.4 ± 14.8	0.61
RAP (mmHg)	7.3 ± 4.9	13.5 ± 5.4	<0.0001
Medical therapy			
Beta blockers	24 (72.7)	52 (69.3)	0.45
ACEI/ARB	23 (69.7)	36 (48.0)	0.03
MRA	6 (18.1)	36 (48.0)	0.003
Loop diuretics	21 (63.6)	63 (84.0)	0.20

ACEI, angiotensin-converting enzyme inhibitor; ARB, angiotensin receptor blocker; CO, cardiac output; eGFR, estimated glomerular filtration rate; Hb, hemoglobin; LVEF, left ventricular ejection fraction; mPAP, mean pulmonary arterial pressure; MRA, mineralocorticoid antagonist; PCWP, pulmonary capillary wedge pressure; PH, pulmonary hypertension; RAP, right atrial pressure; RV, right ventricle; SV, stroke volume.

**Table 2:** Multivariable linear regression analysis adjusted for predictors of last eGFR.

Baseline Parameter	Regression Coefficient (SE)	*t* Value	*p*-Value
Creatinine	−35.74 (4.41)	−8.09	<0.0001
Age (per 10 years)	−7.63 (1.44)	−5.28	<0.0001
Diabetes Mellitus	−8.15 (4.03)	−2.02	0.04
EF < 45%	−18.94 (8.20)	−2.31	0.024
MRA	−10.62 (3.78)	−2.81	0.007

eGFR, estimated glomerular filtration rate; EF, ejection fraction; MRA, mineralocorticoid antagonist; SE, standard error.

**Table 3:** Post-matching multivariable linear regression analysis adjusted for predictors of last eGFR.

Baseline Parameter	Regression Coefficient (SE)	*t* Value	*p*-Value
Creatinine	−36.92 (5.11)	−7.22	<0.0001
Age (per 10 years)	−6.97 (1.53)	−4.55	<0.0001
Diabetes Mellitus	−8.89 (4.66)	−1.91	0.062
EF < 45%	−17.86 (8.69)	−2.05	0.045
MRA	−10.80 (4.29)	−2.52	0.015

eGFR, estimated glomerular filtration rate; EF, ejection fraction; MRA, mineralocorticoid antagonist; SE, standard error.

## Data Availability

The original contributions presented in this study are included in the article. Further inquiries can be directed to the corresponding author.
